# Comparative Chloroplast Genomes of* Sorghum* Species: Sequence Divergence and Phylogenetic Relationships

**DOI:** 10.1155/2019/5046958

**Published:** 2019-03-19

**Authors:** Yun Song, Yan Chen, Jizhou Lv, Jin Xu, Shuifang Zhu, MingFu Li

**Affiliations:** ^1^Institute of Plant Quarantine, Chinese Academy of Inspection and Quarantine, Beijing 100176, China; ^2^Institute of Animal Quarantine, Chinese Academy of Inspection and Quarantine, Beijing 100176, China

## Abstract

Sorghum comprises 31 species that exhibit considerable morphological and ecological diversity. The phylogenetic relationships among S*orghum* species still remain unresolved due to lower information on the traditional DNA markers, which provides a limited resolution for identifying* Sorghum* species. In this study, we sequenced the complete chloroplast genomes of* Sorghum sudanense* and* S. propinquum* and analyzed the published chloroplast genomes of* S. bicolor* and* S. timorense *to retrieve valuable chloroplast molecular resources for* Sorghum*. The chloroplast genomes ranged in length from 140,629 to 140,755 bp, and their gene contents, gene orders, and GC contents were similar to those for other Poaceae species but were slightly different in the number of SSRs. Comparative analyses among the four chloroplast genomes revealed 651 variable sites, 137 indels, and nine small inversions. Four highly divergent DNA regions (*rps16-trnQ*,* trnG-trnM*,* rbcL-psaI*, and* rps15-ndhF*), which were suitable for phylogenetic and species identification, were detected in the* Sorghum* chloroplast genomes. A phylogenetic analysis strongly supported that* Sorghum* is a monophyletic group in the tribe Andropogoneae. Overall, the genomic resources in this study could provide potential molecular markers for phylogeny and species identification in* Sorghum*.

## 1. Introduction

*Sorghum bicolor* (L.) Moench, sorghum, is the fifth in both production and planted area of cereal crops worldwide. It is extensively cultivated in marginal rainfall areas of the tropics and subtropics. The wild species of sorghum represent a potentially diverse source of germplasm for sorghum breeding programs. Sorghum comprises 31 species that exhibit considerable morphological and ecological diversity [1–3]. The genus* Sorghum* has been taxonomically classified into five subgenera or sections:* Chaetosorghum*,* Heterosorghum*,* Parasorghum, Stiposorghum*, and* Sorghum *[3]. Phylogenies based on a sequence analysis suggest that the* Sorghum* subgenera or section designations may not correspond to evolutionary relationships [1, 4, 5]. The phylogenetic relationships within subgenera or sections of* Sorghum* are not clear, and little is known about the phylogenetic relationships among the species.

To determine the phylogenetic relationships of* Sorghum*, molecular markers, including chloroplast genome regions (such as* ndhF*,* psbZ-trnG*,* trnY-trnD*,* trnY-psbM*, and* trnT-trnL*), and multiple nuclear genes (ITS, Pepc4, and GBSSI) have been analyzed [4–9]. However, many relationships within the genus remain unresolved because these markers are of low diversity and only provide a limited resolution for identifying closely related taxa. The development of more effective genetic resources is necessary to infer phylogenic relationships and to identify the species of* Sorghum.*

In recent years, an increasing number of researchers have focused on the chloroplast genome to develop genetic markers for phylogeny and DNA barcoding. In general, chloroplast genomes are in the range of 120-160 kb in length and encode 120 to 130 genes [10]. The chloroplast genome has a conserved quadripartite structure that consists of a large single-copy region (LSC) and a small single-copy region (SSC), which are separated by a pair of inverted repeats (IRs). Moreover, chloroplast genomes are inherited uniparentally (maternally in most angiosperms plants) at a slower evolutionary rate of change compared to nuclear genomes. For these reasons, the chloroplast genome is a potentially useful tool for phylogenetic studies, population genetics, phylogeography, and species identification. Mutations in the chloroplast genome are clustered as mutation hotspots, and this mutational dynamic has resulted in highly variable regions in the genome [11]. Those variable regions are used for phylogeny and species identification [12, 13].

In this study, we sequenced the complete chloroplast genomes of* S. sudanense* and* S. propinquum* which belong to subgenera of* Sorghum* and compared the resulting sequences with the published chloroplast genome of* S. bicolor* [14] and* S. timorense* (GenBank accession number: KF998272). The objective was to compare the chloroplast genomic structure and sequence variation within the genus* Sorghum* to retrieve valuable chloroplast molecular markers for species identification and to clarify the phylogenetic relationship of the tribe Andropogonodae.

## 2. Materials and Methods

### 2.1. Plant Materials, DNA Extraction, and Sequencing

The plant materials of* S. sudanense* and* S. propinquum* were provided by the National Grass Germplasm Bank of China. Fresh leaves from each species were immediately dried with silica gel prior to DNA extraction. The total genomic DNA was isolated from each individual plant using the mCTAB extraction protocol [15] and was purified using the Wizard DNA CleanUp System (Promega, Madison, WI, USA). The total DNA quantity was evaluated by the value of the ratio of absorbance measurements at 260 nm and 280 nm (A260/A280) using NanoDrop 2000 (Thermo Fisher Scientific, Waltham, MA, USA), whereas a visual assessment of the DNA size and integrity was performed using gel electrophoresis. We identified the materials using the ITS sequences. The ITS sequencing methods followed Ng'uni et al. [6] and the ITS sequences were submitted to GenBank (accession numbers: MK514589 and MK514590).

The chloroplast genomes of* S. sudanense* and* S. propinquum* were sequenced using the long-range PCR method reported by Dong* et al*. [10]. The PCR protocol was as follows: preheating at 98°C for 2 min, 40 cycles at 98°C for 10 s, annealing at 50°C for 30 s, and elongation at 72°C for 5 min, followed by a final extension at 72°C for 10 min. PCR amplification was performed in an Applied Biosystems Veriti™ 96-Well Thermal Cycler (Model #: 9902, made in Singapore).

PCR products were randomly fragmented into 400–600 bp using an ultrasonicator. An Illumina paired-end DNA library with a 500 bp insert size was constructed using a NEBNext® Ultra™DNA Library Prep Kit following the manufacturer's instructions. The library was sequenced by the Illumina Hiseq X Ten platform double terminal sequencing method.

### 2.2. Assembly and Annotation

The paired-end reads were qualitatively assessed and were assembled using SPAdes 3.6.1 [16]. Chloroplast genome sequence contigs were selected from the initial assembly by performing a BLAST search using the* Sorghum bicolor* chloroplast genome sequence as a reference (GenBank accession number: EF115542). The selected contigs were assembled with Sequencher 5.4.5 (http://www.genecodes.com). The gaps and ambiguous sequences were manually adjusted after Sanger sequencing. PCR amplification and Sanger sequencing were performed to verify the four junction regions between the IRs and the LSC/SSC [17]. The chloroplast genome annotation was performed with Plann [18] using the* Sorghum bicolor *reference sequence from GenBank. The chloroplast genome map was drawn using Genome Vx software [19].

### 2.3. Simple Sequence Repeat (SSR) Analysis

Perl script MISA (http://pgrc.ipk-gatersleben.de/misa/) was used to detect the chloroplast simple sequence repeats in four chloroplast genome sequences of* Sorghum*. Its parameters were set as follows: the minimum numbers of repeats for mononucleotide, dinucleotides, trinucleotides, tetranucleotides, pentanucleotide, and hexanucleotides were 10, 5, 4, 3, 3, and 3, respectively. At the same time, the SSR of the IR, LSC, SSC, and coding regions, introns, and intergenic regions that correspond to different regions were analyzed.

### 2.4. Variation Analyses

All sequenced* Sorghum* chloroplast genomes were aligned using MIFFT v7 [20]. SNPs and the microstructure (indels and inversions) were checked in the four* Sorghum* chloroplast genomes. The SNPs were calculated using MEGA 6.0 [21]. Based on the aligned sequence matrix, the indel events were checked manually and were further divided into two categories: microsatellite-related indels (SSR-indel) and non-microsatellite-related indels (NR-indel).

Using the* S. sudanense* chloroplast genome sequence as the standard reference, the size, location, and evolutionary direction of the microstructure events were counted. The proposed secondary structures of the inverted regions were analyzed using mfold software [22].

### 2.5. Molecular Marker Development

A sliding window analysis was conducted to generate nucleotide diversity of the chloroplast genome using DnaSP v5.10 software [23]. The step size was set to 100 bp with a 600 bp window length.

### 2.6. Phylogenetic Reconstruction

To investigate the phylogenetic position of* Sorghum*, we used 41 complete chloroplast genomes (Table S1). Among them, 36 were from Andropogonodae, and five other species from different tribes (*Garnotia tenella, Centotheca lappacea*,* Chasmanthium laxum*,* Gynerium sagittatum*, and* Pseudolasiacis leptolomoides*) were used as the outgroups. Sequence alignments were carried out using MIFFT v7 [20] and then were adjusted manually using Se-Al 2.0. [24].

Phylogenetic analyses were conducted using the maximum likelihood (ML) and the Bayesian inference (BI) methods. The ML analysis was conducted using RAxML version 8.0.20 with 500 bootstrap replicates. The GTRGAMMA model was used in all of the ML analyses as is suggested in the RAxML manual.

MrBayes 3.2.2 [25] was used to perform a Bayesian inference analysis. The Markov chain Monte Carlo (MCMC) analysis was run for 2 × 5,000,000 generations. The average standard deviation of split frequencies remained below 0.01 after the fifty percent burn-in. The remaining trees were used to build a 50% majority-rule consensus tree.

## 3. Results and Discussion

### 3.1. Features of* Sorghum* Chloroplast Genomes

The plastomes of the four species contain no significant differences in their contents of genes and introns, the gene order in the four genomes is identical, and the sizes of LSC, SSC, and IR regions are very similar. The overall GC content of the chloroplast genome is 38.5%, which is consistent with reported Poaceae species [12, 26].

**Figure 1:**
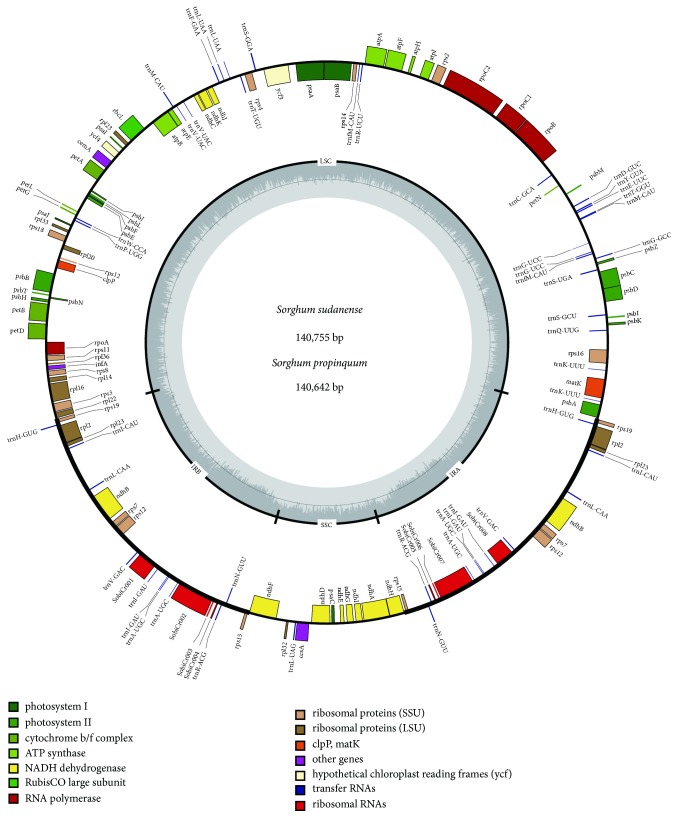
*Map of the Sorghum chloroplast genome.* The genes inside and outside the circle are transcribed in the clockwise and counterclockwise directions, respectively. Genes belonging to different functional groups are shown in different colors. Thick lines indicate the extent of the inverted repeats (IRa and IRb) that separate the genomes into small single-copy (SSC) and large single-copy (LSC) regions.

A total of 110 unique genes were identified in the* Sorghum* chloroplast genome, including 77 protein-coding genes, 29 tRNA genes, and 4 ribosomal RNA genes (Figure 1, Table 1 and Table S2). Notably, seven protein-coding genes (*rps15*,* rps12*,* rps7*,* ndhB*,* rpl23*,* rpl2,* and* rps19*) eight tRNA genes (*trnA-UGC*,* trnH-GUG*,* trnI-CAU*,* trnI-GAU*,* trnL-CAA*,* trnN-GUU*,* trnR-ACG*, and* trnV-GAC*), and all of the rRNA genes are duplicated in the IR regions, which is common in most Poaceae genomes. In the* Sorghum* chloroplast genome, there were 18 intron-containing genes. Among them, ten protein-coding genes (*petB*,* petD*,* atpF*,* ndhB*,* ndhA*,* rpoC1*,* rps12*,* rps16*,* rpl16*, and* rpl2*) and six tRNA genes have a single intron and two genes (*clpP* and* ycf3*) that contained two introns. The* rps12* is a trans-splicing gene, with the 5′ end located in the LSC region and the duplicated 3′ end located in the IR region. The* matK *was located within the intron of* trnK-UUU*.

### 3.2. Simple Sequence Repeats

Simple sequence repeats (SSRs) are a type of 1–6 nucleotide unit tandem repeat sequence that is frequently observed in chloroplast genomes. These are important molecular markers for plant population genetics, evolution, and ecological studies because of their high diversity in copy numbers within species due to slipped strand mispairing during DNA replication on a single DNA strand [27, 28].

**Figure 2:**
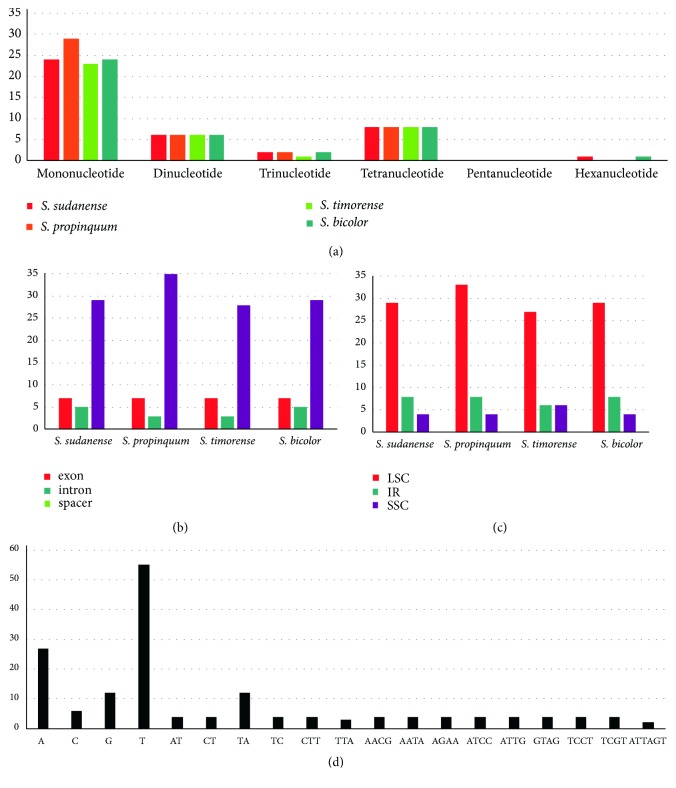
*Analyses of simple sequence repeat (SSR) in the four Sorghum chloroplast genomes.* (a) Number of different SSRs types detected by MISA; (b) number of SSRs in LSC, SSC, and IR regions; (c) number of SSRs in spacer, exon, and intron; (d) frequency of identified SSR motifs in the different repeat classes.

There were 38, 41, 41, and 45 simple sequence repeats in the chloroplast genomes of* S. timorense*,* S. bicolor*,* S. sudanense*, and* S. propinquum*, respectively (Figure 2, Table S3). The mononucleotide SSRs were the richest, with a proportion of 60.61%, followed by dinucleotide SSRs (14.55%), tetranucleotide SSRs (19.39%), and trinucleotide SSRs (4.24%). One hexanucleotide SSR was found in* Sorghum sudanense* and* Sorghum bicolor*. Pentanucleotide was not detected in the Sorghum chloroplast genomes. The majority of SSRs in all species were A/T mononucleotides. Chloroplast genome SSRs were composed of adenine or thymine repeats and rarely contained tandem guanine (G) or cytosine (C) repeats. The majority of SSRs were located in the LSC region (71.52). Furthermore, most of the SSRs were found in space regions (73.33%), followed by exon regions (16.97%) and intron regions (9.70%). SSRs in the chloroplast genome have been shown to be extremely useful for resolving genetic diversity between closely related taxa and, hence, increase the power of interspecific studies [29, 30], possibly in combination with other informative nuclear genome SSRs.

### 3.3. Numbers and Pattern of SNP Mutations

In total, 651 single nucleotide substitutions (SNP) were detected in the four* Sorghum* chloroplast genomes, 518 of which were found in the LSC region, 18 in the IR region, and 97 in the SSC region. The number of SNP among the four* Sorghum *species was found to be 3 to 631.* S. timorense *exhibits higher divergence than other three species.* S. sudanense *and* S. bicolor* show the lowest sequence divergence.

**Figure 3:**
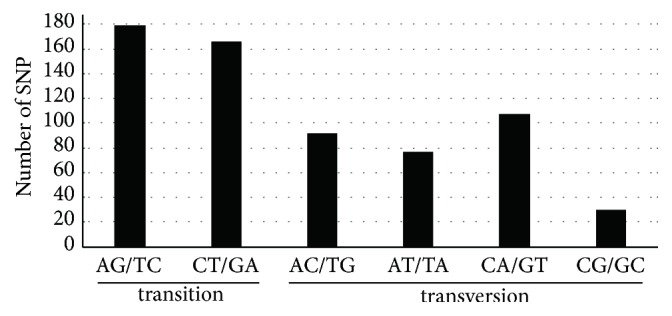
*The patterns of nucleotide substitutions among the four Sorghum chloroplast genomes.* The patterns were divided into six types as indicated by the six non-strand-specific base-substitution types (i.e., numbers of considered G to A and C to T sites for each respective set of associated mutation types).

The pattern of SNP mutation is shown in Figure 3. There were 345 transitions (Ts) and 306 transversions (Tv) and the Tv to Ts ratio was 1:0.89, which indicated a bias in favor of transitions. The most frequently occurring mutations were from A to G and from T to C substitutions (179), while from C to G and from G to C exhibited the lowest frequency (30). Despite the higher A+T contents in chloroplast genomes, AT to TA transversions among the four types of transversions were found to occur significantly less frequently (Figure 3). It is clear that there is a bias in the chloroplast genomes [31].

### 3.4. Indels

There were 137 indels in the chloroplast genome, which was identified among the four* Sorghum* chloroplast genomes (Tables S4 and S5), including 43 indels that are caused by SSR variations (SSR-indels) and 94 non-SSR-related indels (NR-indels). The majority of SSR-indels were related to A/T types SSRs (39 times). Only one dinucleotide SSR indel was identified, which is located in* ndhF-rpl32*. All of the SSR-indels were found in the noncoding regions of the LSC/SSC section.

**Figure 4:**
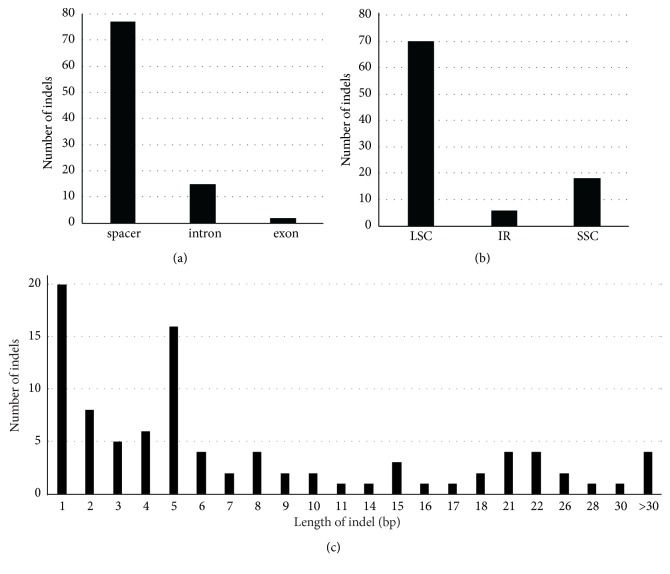
*NR-indels identified in the chloroplast genomes of the four Sorghum chloroplast genomes*. (a) Numbers of individual NR-indels shown by sequence length; (b) relative frequency of NR-indel occurrence in LSC, SSC, and IR regions.

The size of NR-indels ranged from 1 to 165 bp, with one bp long indel and 5 bp long indels being the most common (Table S5 and Figure 4). The largest one, found in* rpoC* with 165 bp length, was a deletion in the* S. sudanense*. The second longest, which was found in* rps16-trnQ *with 152 bp length, was an insertion in* S. timorense*. Finally, 46 insertion indels and 42 deletion indels were specific to* S. timorense*, one insertion indel and two deletion indels were specific to* S. sudanense*, and one insertion in* rpoC1* intron was specific to* S. propinquum. *Most of the NR-indels were located in noncoding regions (81.91% in space and 15.96% in introns).

Indels were another important class of genetic variation compared with nucleotide substitutions. Several molecular processes are known to create indels. Polymerase slippage processes during DNA replication or repair can result in the addition or deletion of short spans of sequence that repeat at one side of the region flanking the indel [32], which mainly created SSR-indel type. SSR-indels in chloroplast genome were primarily found in AT-regions and often involve long stretches of repeats of a single nucleotide [33]. In the* Sorghum* chloroplast genome, most of the SSR-indels (90.70%) were A/T types. Hairpins or the stem-loop secondary structure and intramolecular recombination are thought to cause the majority of NR-indel mutations [33]. Different types of indels also show varying amounts of homoplasy. SSR-indels seem to be more prone to homoplasy between different species [28, 34]. In this study, NR-indels were often less homoplasious (Table S5). An increasing number of studies have shown that indel characters can be extremely useful for inferring relationships among more closely related taxa [30, 35, 36].

### 3.5. Small Inversions

Nine small inversions of 2 to 6 bp were identified in the* Sorghum* chloroplast genomes (Table 2). Eight inversions occurred in the LSC region, and one occurred in the SSC region. Most of the small inversions are in intergenic spacer regions, with only two exceptions. One is a 4 bp inversion within the coding region of* ccsA*, and the other is a 4 bp inversion in the* rpl16* intron. All of the inversions and their inverted repeating flanking sequences can form stem-loop structures. The franking repeats are from 3 to 20 bp in length. All inversions occurred in* S. timorense* except the inversion in ccsA, which occurred in* S. sudanense*.

Many small inversions may have been generated by parallel or back mutation events during chloroplast genome evolution [37, 38]. However, recent studies suggest that, at least in some groups, some small inversions are valuable for a phylogenetic relationship [34]. All of the small inversions in the four* Sorghum* chloroplast genomes had phylogenetic information.

### 3.6. Divergent Hotspots

**Figure 5:**
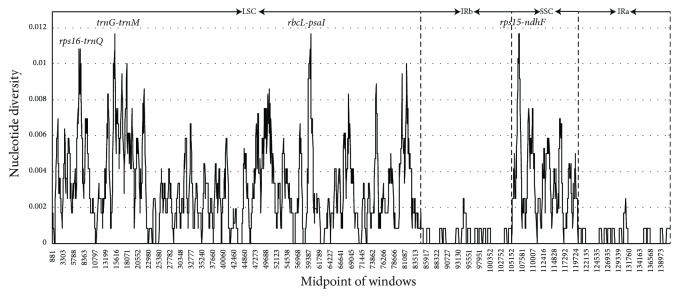
*Sliding window analysis of the Sorghum chloroplast genomes.* X-axis: position of the midpoint of a window; Y-axis: nucleotide diversity of each window.

Divergent hotspots in the chloroplast genomes between different species at the genus level have provided abundant informative loci for systematic plant and DNA barcoding research [11, 39, 40]. Furthermore, a sliding window analysis using DnaSP detected highly variable regions in the* Sorghum *chloroplast genome. Nucleotide diversity values within 800 bp varied from 0 to 0.01167, and the average value of PI was 0.00965. The IR regions exhibited lower variability than the LSC and SSC regions (Figure 5). There were four mutational hotspots that showed remarkably higher PI values (>0.01), including three intergenic regions (*rps16-trnQ*,* trnG-trnM*, and* rbcL-psaI*) in the LSC and one intergenic region (*rps15-ndhF*) in the SSC from the chloroplast genomes.

*Rps16-trnQ* are highly variable in most plant groups and have been used in previous phylogenetic studies [11, 41–43]. In Veroniceae,* trnG-trnM* was also identified as a highly variable locus [44]. K Saltonstall [45] provided a set of primers to amplify the* rbcL-psaI* region in the grass. The* rbcL-psaI *has been used for phylogeographic inference of* Phragmites australis* [46].* rps15-ndhF* combined with five other chloroplast markers has been used to successfully resolve relationships and investigate the biogeography in woody bamboos (Poaceae: Bambusoideae) [47]. These four mutation “hotspot” regions could provide adequate genetic information for* Sorghum* species identification and phylogeny analysis.

### 3.7. Phylogenetic Analysis

Chloroplast genome sequences have been successfully used for the reconstruction of phylogenetic relationships among plant lineages [48–51]. Phylogenetic analyses of plant species using a small number of loci might frequently be insufficient to resolve evolutionary relationships, particularly at low taxonomic levels [52, 53]. Much of the previous phylogenetic work based on whole chloroplast genomes has been used to resolve difficult phylogenetic relationships among closely related species [40, 54].

**Figure 6:**
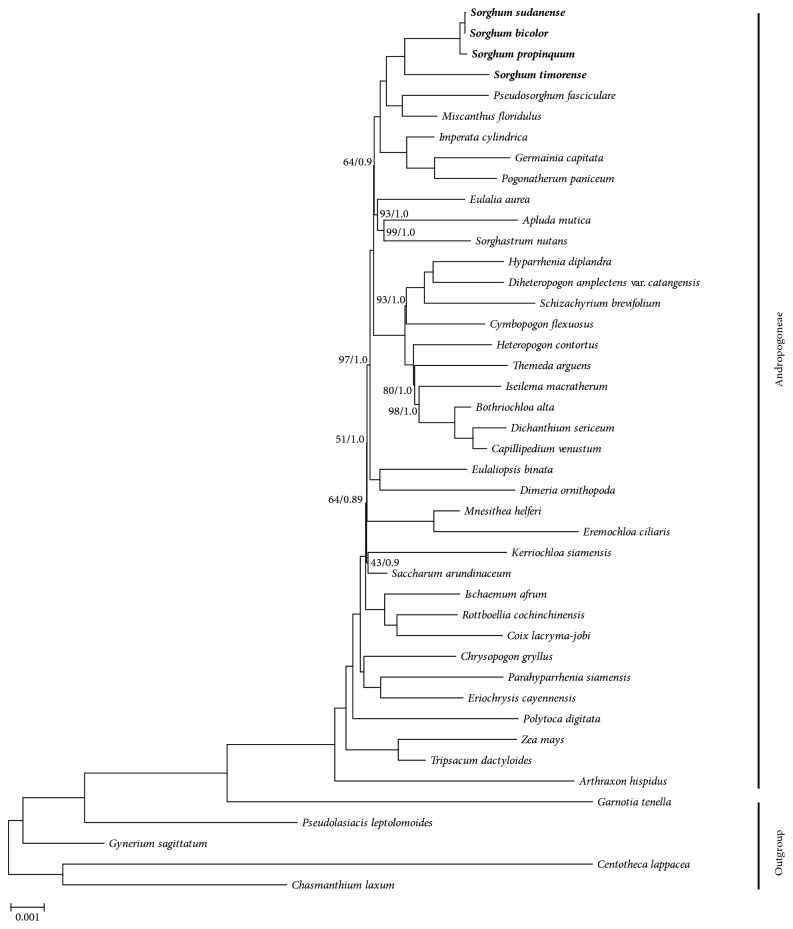
*Phylogenetic relationships of the Andropogoneae species constructed from the complete chloroplast genome sequences using maximum likelihood (ML) and Bayesian inference (BI).* ML topology shown with ML bootstrap support value (BP)/Bayesian posterior probability (PP) given at each node. Nodes with 100 BP/1.0 PP are not marked.

To understand the evolution of Andropogoneae, an improved resolution of phylogenetic relationships has been achieved using the fully sequenced chloroplast genome sequences of 38 Andropogoneae species (Figures 6 and S1). The maximum likelihood (ML) and Bayesian inference (BI) trees exhibited similar phylogenetic topologies. The phylogenetic analyses supported the monophyly of Andropogoneae with strong bootstrap support (BS) of 100% and posterior probabilities (PP) of 1.0 and contributed to clarifying intergeneric relationships (Figures 6 and S1).* Arthraxon *was well resolved as the first-branching lineage (BS=100; PP=1.0). The short branch lengths in some nodes of the tree suggested the rapid radiation evolutionary history in these clades. Skendzic et al. [55] used ITS and* trnL–F* to investigate the phylogeny of Andropogoneae; the result showed that most of Clayton and Renvoize's [56] subtribes are not monophyletic. Using the chloroplast genome dataset, this study inferred the clear relationship of Andropogoneae, and this result is consistent with Skendzic et al. 's.

*Sorghum* was a monophyletic sister to* Pseudosorghum *and* Miscanthus* (BS=100, PP=1.0). The four* Sorghum *species were grouped into two groups.* S. sudanense, S. bicolor*, and* S. propinquum *formed a group.* S. sudanense, S. bicolor*, and* S. propinquum *belong to the subgenus* Sorghum *which contain ten species. The phylogeny of subgenus* Sorghum* was unclear because of the low divergence among those species. Several studies used chloroplast markers (*ndhF*,* psbZ-trnG*,* trnY-trnD*,* trnY-psbM*, and* trnT-trnL*) and nuclear markers (ITS, Pepc4, and GBSSI) to infer the phylogeny of* Sorghum *[4–6, 8]. Those results supported that* S. sudanense, S. bicolor*, and* S. propinquum *formed a group.* S. sudanense *is believed to be segregate from a natural hybrid between* S. bicolor* and* S. arundinaceum *[57]. This is consistent with the present results, which place* S. sudanense* in close relationship with* S. bicolor* with 100% support (Figure 6).

Therefore, it is crucial to use more species to better understand Andropogoneae and* Sorghum* phylogeny and evolution. This study provides a basis for the future phylogenesis of Andropogoneae species.

## Figures and Tables

**Table 1:** Details of the complete chloroplast genomes of the four *Sorghum *species.

Genome features	*S. sudanense*	*S. propinquum*	*S. bicolor*	*S. timorense*
Size (bp)	140755	140642	140754	140629
LSC length (bp)	82686	82572	82685	82587
IR length (bp)	22783	22782	22783	22752
SSC length (bp)	12503	12506	12503	12538
Total genes	110	110	110	110
Protein coding genes	77	77	77	77
tRNA genes	29	29	29	29
rRNA genes	4	4	4	4
Overall GC content (%)	38.5	38.5	38.5	38.5
Accession number in GenBank	MH926028	MH926027	EF115542	KF998272

**Table 2:** The location, direction, and length of nine small inversions in the four *Sorghum *chloroplast genomes.

	Length of inversions (bp)		Direction of the small inversions			
Location	Length of inversion	Length of inverted repeat	*S. sudanense*	*S. propinquum*	*S. bicolor*	*S. timorense*
*rps16-trnQ*	2	12	no	no	no	yes
*trnT-trnE*	2	6	no	no	no	yes
*trnT-trnE*	2	5	no	no	no	yes
*psbM-petN*	6	14	no	no	no	yes
*rbcL-rpl32*	6	8	no	no	no	yes
*petA-psbJ*	6	14	no	no	no	yes
*rpl33-rps18*	2	3	no	no	no	yes
*rpl16* intron	4	8	no	no	no	yes
*ccsA*	4	20	no	yes	yes	yes

## Data Availability

The sequences of* Sorghum propinquum* and* Sorghum sudanense* chloroplast genome are deposited in the GenBank of NCBI under Accession nos. MH926027 and MH926028. The ITS sequences of* S. sudanense* and* S. propinquum* were available in GenBank database under Accession nos. MK514589 and MK514590.

## Supplementary Materials

Supplementary 1 Table S1: accession numbers and relevant references of the sampled Andropogonodae chloroplast genome sequences obtained from GenBank in this study.

Supplementary 2 Table S2: list of genes present in the Sorghum chloroplast genome.

Supplementary 3 Table S3: SSR loci identified in the four* Sorghum* chloroplast genomes. Positions, locations, types, and polymorphisms are shown.

Supplementary 4 Table S4: polymorphic SSR-indels identified in the analyzed material. For each species, the number of repeats for each SSR is provided.

Supplementary 5 Table S5: polymorphic NR-indels identified in the analyzed material. Indel events are reported for each of the four* Sorghum* species.

Supplementary 6 Figure S1: phylogenetic relationships of the Andropogoneae species constructed from the complete chloroplast genome sequences using Bayesian inference (BI).
