# Supplementary material for: Comparative Chloroplast Genomes of Sorghum Species: Sequence Divergence and Phylogenetic Relationships
Source: Biomed Res Int. 2019 Mar 19;2019:5046958. doi: 10.1155/2019/5046958 (PMC6444266; doi:10.1155/2019/5046958)

Figure S1. Phylogenetic relationships of the Andropogoneae species constructed from the complete chloroplast genome sequences using Bayesian inference (BI).

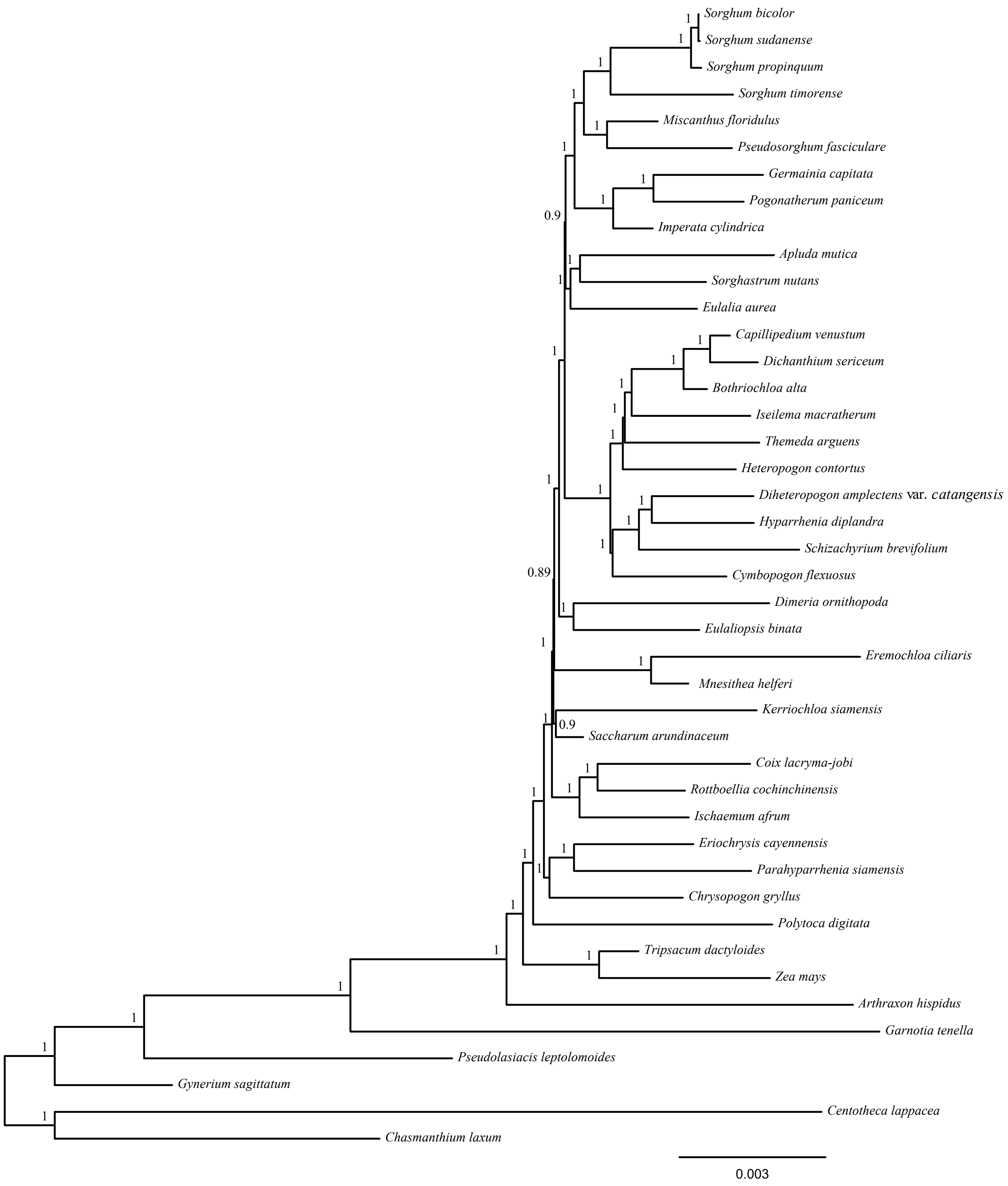

Supplement: Supplementary 6 — Figure S1: phylogenetic relationships of the Andropogoneae species constructed from the complete chloroplast genome sequences using Bayesian inference (BI). [file 5046958.f6.pdf]
